# Heat transfer analysis of Maxwell tri-hybridized nanofluid through Riga wedge with fuzzy volume fraction

**DOI:** 10.1038/s41598-023-45286-x

**Published:** 2023-10-25

**Authors:** Rana Muhammad Zulqarnain, Muhammad Nadeem, Imran Siddique, Hijaz Ahmad, Sameh Askar, Mahvish Samar

**Affiliations:** 1 School of Mathematical Sciences, Zhejiang Normal University, Jinhua, 321004 Zhejiang China; 2 Department of Mathematics, University of Management and Technology, Lahore, 54770 Pakistan; 3 Section of Mathematics, International Telematic University Uninettuno, Corso Vittorio Emanuele II, 39, 00186 Roma, Italy; 4 Operational Research Center in Healthcare, Near East University, Near East Boulevard, 99138 Nicosia/Mersin 10, Turkey; 5 Department of Statistics and Operations Research, College of Science, King Saud University, P.O. Box 2455, Riyadh, 11451 Saudi Arabia; 6 School of Computer Science and Technology, Zhejiang Normal University, Jinhua, 321004 China

**Keywords:** Mathematics and computing, Physics

## Abstract

This contribution aims to optimize nonlinear thermal flow for Darcy-Forchheimer Maxwell fuzzy $\left( {{\text{Al}}_{{2}} {\text{O}}_{{3}} + {\text{Cu }} + {\text{TiO}}_{{2}} {\text{/EO}}} \right)$ tri-hybrid nanofluid flow across a Riga wedge in the context of boundary slip. Three types of nanomaterials, ${\text{Al}}_{{2}} {\text{O}}_{{3}} ,$ Cu and ${\text{TiO}}_{2}$ have been mixed into the basic fluid known as engine oil. Thermal properties with the effects of porous surface and nonlinear convection have been established for the particular combination $\left( {{\text{Al}}_{{2}} {\text{O}}_{{3}} + {\text{Cu}} + {\text{TiO}}_{{2}} {\text{/EO}}} \right){.}$ Applying a set of appropriate variables, the set of equations that evaluated the energy and flow equations was transferred to the dimensionless form. For numerical computing, the MATLAB software's bvp4c function is used. The graphical display is used to demonstrate the influence of several influential parameters. It has been observed that flow rate decay with expansion in porosity parameter and nanoparticles volumetric fractions. In contrast, it rises with wedge angle, Grashof numbers, Darcy-Forchheimer, nonlinear Grashof numbers, and Maxwell fluid parameter. Thermal profiles increase with progress in the heat source, nanoparticles volumetric fractions, viscous dissipation, and nonlinear thermal radiation. The percentage increases in drag force for ternary hybrid nanofluid are 13.2 and 8.44 when the Modified Hartmann number takes input in the range $0.1 \le {\text{Mh}} \le 0.3$ and wedge angle parameters $0.1 \le m \le 0.3$. For fuzzy analysis, dimensionless ODEs transformed into fuzzy differential equations and employed symmetrical triangular fuzzy numbers (TFNs). The TFN makes a triangular membership function (M.F.) that describes the fuzziness and comparison. This study compared nanofluids, hybrid nanofluids, and ternary nanofluids through triangular M.F. The boundary layer flow caused by a wedge surface plays a crucial role in heat exchanger systems and geothermal.

## Introduction

Non-Newtonian fluids are complicated fluids that a solitary relationship cannot represent. Many applications, such as ketchup, sugar solutions, apple sauce, starch suspensions, soaps, lubricants, and margarine, can display non-Newtonian fluid characteristics. A rate-type subclass called the Maxwell fluid (viscous and elastic behavior) is a non-Newtonian representation that displays stress relaxation behaviors. Scientists are eager to discover more about its distinctive qualities. Because of the broad array of uses that eventuate in engineering, industry, and natural processes such as cooling of electronic equipment, human transpiration, some biological fluids, DNA suspension, escalation of chemicals in plants or medicine, aerodynamic extrusion, and many others, it has gained remarkable significance and expanded motivations or consciousness between many scientists to do work in the area over the last couple of years. Well-known researchers in recent research work have made some remarkable contributions towards Maxwell nanofluid. Bilal et al.^1^ examined the radiation heat flux of MHD Maxwell fluid over an upper-connected surface. Jamshed et al.^2^ studied the various aspects of MHD Maxwell nanofluids. The chemical reactions on Maxwell flow via permeable surface under the effect of radiations and multiple slips have been inspected by Ali et al.^3^. Abdal et al.^4, 5^ reported PST and PHF properties on MHD Maxwell fluid-containing living organisms. Bilal et al.^6^ analyzed the numerical simulation of the time-dependent Maxwell flow of nanofluids inspired by melting heat, magnetic fields, and Fourier and Fick laws. The liquid temperature drops when compared to melting heat and unsteadiness parameters, according to this study. Tlili et al.^7^ evaluated the flow of a 3D solutal and thermal stratification on Maxwell nanofluid with a chemical process. Yahya et al.^8^ studied the heat transfer analysis on Maxwell hybrid $\left( {{\text{SiO}}_{{2}} + {\text{TiO}}_{{2}} {\text{/ Kerosene oil}}} \right)$ nanofluid over a Riga wedge with a viscous dissipation effect. Babitha et al.^9^ estimated the flow of MHD on fractional Maxwell nanofluids suspended with SWCNT and MWCNT. Several investigators have disclosed various characteristics of the Maxwell liquid^10, 11^ and^12^.

Darcy's law, which describes how liquid flows via a porous channel, is when Darcy-Forchheimer gets its name. This law was established based on the findings of an investigation of flowing water over the sand layer. Reynolds number variations in the porous medium, distinguished by strong inertial forces, cause movement. The significant usage of the Darcy law in petroleum technologies, grain storage, groundwater, and oil asset contexts makes it essential to study fluid mechanics. In 1856, Darcy^13^ was the first researcher to claim that liquid may pass through a porous surface. Unfortunately, this idea could not be as well-known due to its limits of a slower pace and lower porosity. Forchheimer^14^ changed the equation of motion by substituting the quadratic velocity requirement with the Darcian velocity to show the obvious deficiency. The high Reynolds number led Muskat^15^ to coin the phrase "Forchheimer word" to describe it. To implement the Darcy-Forchheimer model to permeable media outside of the linearly enlarged zone, Pal and Mondal^16^ assumed that as the electric field value increases, the concentration distribution diminishes. The flow of the MHD nanofluid via the Darcy-Forchheimer media platform as a result of the second-order boundary condition is computed by Ganesh et al.^17^. Darcy–Forchheimer law, homogeneous/heterogeneous reactions, and carbon nanotubes were used by Alshomrani et al.^18^. The Darcy-Forchheimer effect was assessed over a curving surface by Saif et al.^19^. Seth et al.^20^ computed the flow of carbon nanotubes in a moving frame across a porous Darcy–Forchheimer environment. Several researchers have worked on Darcy–the Forchheimer law in the references^21, 22^ and^23^.

The insert of nanoparticles into a base fluid has revolutionized the field of fluid dynamics. Single-type nanoparticles are combined with the base fluid to create a nanofluid. Water, oils, ethylene glycol, and synthetic fluids are commonly used to disseminate nanoparticles. These fluids are not capable of improving heat transfer as compared to nanofluids. Metallic nanoparticles such as silver, gold, titanium, iron oxide, and aluminum are widely utilized in the base fluid. These fluids are engaged as coolants in heat transfer equipment like pharmaceutical processes, heat exchangers, engines, power plants, radiators, and electrical devices, among other things. Choi^24^ first used nanoparticle dispersion in a host fluid to improve its thermal characteristics. Shafiq et al.^25^ and Siddique et al.^26, 27^ have significantly improved base fluids' thermal properties. A hybrid nanofluid is a solution of two different types of nanoparticles in a base fluid with a higher thermal conductivity. Hybrid nanofluids simultaneously increase the chemical and physical properties of constituents. Compared to ordinary fluids and nanofluids, hybrid nanofluids significantly impact optimizing heat transfer since they have higher thermal efficiency and can be molded to meet specific needs. The effects of heat-resistive flow on MHD Sakiadis hybrid nanofluid flow through a thin needle were examined by Tlili et al.^28^. Roy et al.^29^ tested the particular solution of Stoke's second theorem with the flow of a hybrid nanofluid. Shehzad et al.^30^ evaluated heat conduction in a radiative hybrid nanofluid through a permeable zone. Acharya-et al.^31^ explored the role of radiation on hybrid nanofluid $\left( {{\text{Fe}}_{{3}} {\text{O}}_{{4}} {\text{/graphene}}} \right)$ flow via a folded surface. Said et al.^32^ reviewed the newest advances in solar thermal collectors, emphasizing the use of nanofluids as heat transfer fluids to improve the devices' performance. Said et al.^33^ examined current developments in phase change material nanotechnology with an emphasis on solar energy applications. The thermal properties of a Williamson hybrid nanofluid (MoS2 + ZnO) based on hydraulic fluid flowing across a stretched sheet were examined by Yahya et al.^34^. The numerical treatment of fuzzy hybrid nanofluid flow and heat transfer over a surface was investigated by Nadeem et al.^35, 36^.

Ternary hybrid nanofluid is a novel type of fluid that leads regular fluids, nanofluid, hybrid nanofluid, acetone, and gasoline at energy exchanges. Ternary Hybrid NFs are used in heat pumps, solar energy, heat exchangers, the auto industry, air purifiers, electrical chillers, broadcasters, ships, turbines, nuclear networks, and biotechnology. Adun et al.^37^ discussed ternary hybrid nanofluid's stability, heat transfer, environmental factors, and synthesis. Sundar et al.^38^ addressed irreversibility generation and heat transport in an ethylene glycol-based ternary hybrid nanofluid. Ahmed et al.^39^ considered the rise of heat transmission for ternary hybrid nanofluid over a square channel. Their findings proved that ternary hybrid nanofluids are better for thermophoresis and nano-cooling growth. Arif et al.^40^ considered the performance of heat transmission and flow for various shaped nanoparticles based on ternary hybrid nanofluids. Gul and Saeed^41^ addressed enhancing the thermal flow of the couple stress ternary hybrid $\left( {{\text{TiO}}_{{2}} + {\text{CoFe}}_{{2}} {\text{O}}_{{4}} + {\text{MgO/H}}_{{2}} {\text{O}}} \right)$ nanofluid over a nonlinear stretching sheet with the Darcy–Forchheimer law.

In dynamical systems, different kinds of fuzziness or uncertainties happen related to measuring errors, material properties, environmental factors, incomplete knowledge, dimensional tolerances, comparison, engineering parameters, initial and boundary conditions, etc. This fuzziness or uncertainties will undoubtedly affect the dynamic systems, which might change the result because of the dynamic responses. In fluid dynamics, the engineering and heat transfer parameters, like nanoparticle volume fraction, exist in the governing equations. These are neither measured exactly nor their specific nominal values. So, in actual practice, these values are fuzzy or uncertain because their given information is incomplete, vague, or imprecise. In this situation, fuzzy sets theory (FST) is a useful tool for the phenomena under consideration, and it is more accurate than assuming physical difficulties. More precisely, the FDEs play a significant role in reducing the uncertainty and finding the proper way to describe the physical problem that arises in uncertain heat transfer parameters initial and boundary conditions. In 1965, Zadeh^42^ gave an alternate idea of set theory, which is named FST, and this approach handles imprecise or uncertain information. The notion of fuzzy number (FN) was presented by Chang and Zadeh^43^. Further, these numbers were generalized by Dubois and Prade^44^. Different types of FNs can be categorized as triangular, trapezoidal, and Gaussian fuzzy numbers. Here, we consider TFNs for the sake of completeness. In 1987, Seikkala^45^ introduced the concept of fuzzy differentiability. Later on, Kaleva^46^ presented fuzzy differentiation and integration. Kandel and Byatt^47^ introduced the FDEs in 1987. Several researchers have used FDEs in fluid mechanics and have found positive results. Nadeem et al.^48^ investigated fuzzy ohmic heating on the MHD third-grade fluid in an inclined channel. Poiseuille and Couette flows were used to study third-grade fluid in a fuzzy atmosphere scrutinized by Nadeem et al.^49^. The MHD flow of the third-grade fluid across two parallel plates in a fuzzy environment was reported by Siddique et al.^50^. In their study, Nadeem et al.^49^ attempted to understand how third-grade fluids behaved in Couette and Poiseuille flow when placed in a fuzzy environment. The MHD, steady, and mixed convection flow through a vertical wedge with porous material was analyzed by Kumari et al.^51^. The 2D time-dependent nanofluid flow via a vertical wedge and nonlinear mixed convection has been examined by Rajput et al.^52^.

The subsequent points demonstrate the originality of this article:

i. The thermal flow rate is inflated by suspending three distinct ${\text{Al}}_{{2}} {\text{O}}_{{3}} {\text{, Cu, and TiO}}_{{2}}$ nanoparticles in Engine oil.
ii. The transportation of nanofluids takes place through the Riga wedge, and its boundary is slipped and convected.
iii. For the flow system, a Darcy-–Forchheimer for the porous medium is examined.
iv. The Maxwell fluid is applied.
v. Nonlinear forms of thermal radiation and convection are analyzed.
vi. For the fuzzy analysis, the nanoparticle volume fractions are said to be TFN controlled $\sigma {\text{ - cut}}{.}$ Also, the comparison of the nanofluid, hybrid, and ternary hybrid nanofluid is examined.

## Mathematical formulation

**Figure 1:**
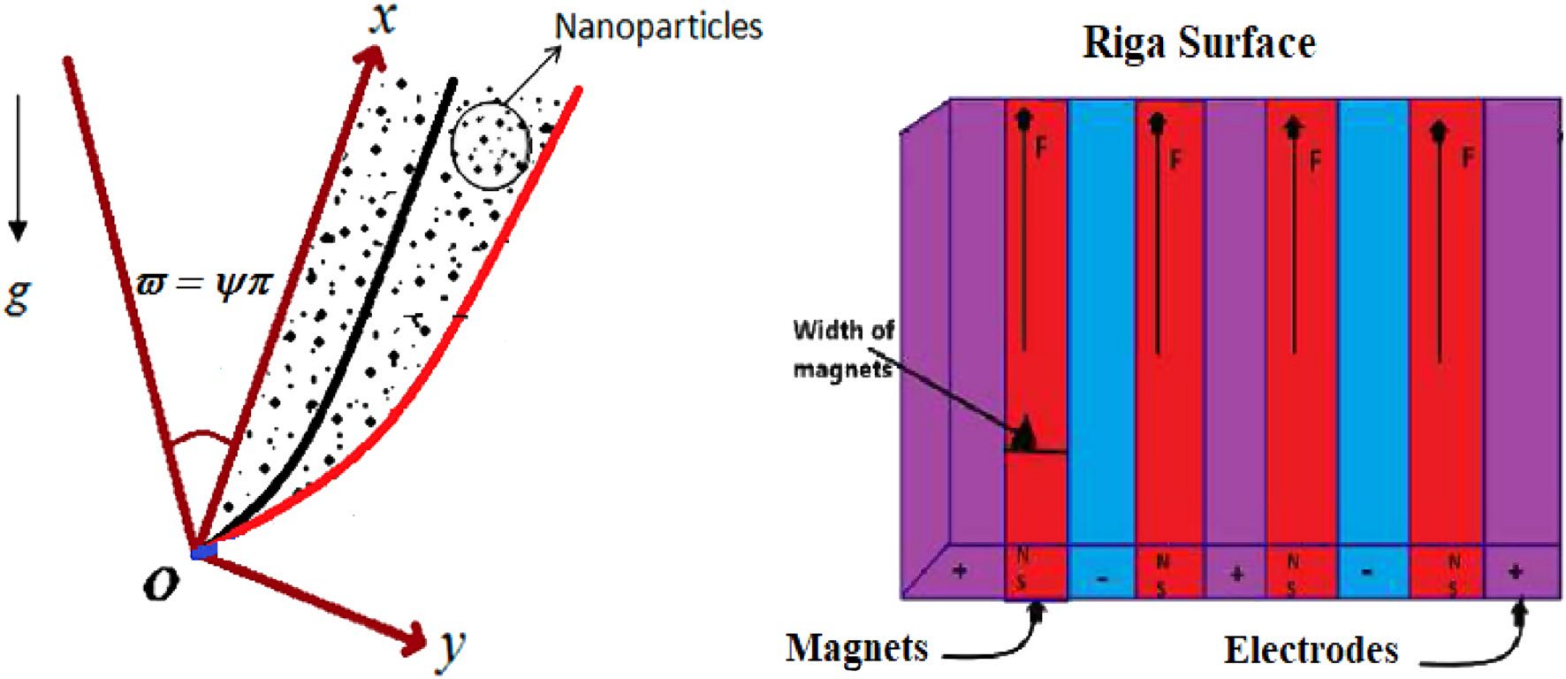
Physical flow model.

Assume that steady, 2D, nonlinear mixed convection and thermal radiation on a Maxwell ternary hybrid nanofluid across a vertical wedge with Darcy–Forchheimer law. The ternary hybrid nanofluid $\left( {{\text{Al}}_{{2}} {\text{O}}_{{3}} {\text{ + Cu}} + {\text{TiO}}_{{2}} {\text{/EO}}} \right)$ flows on stream coordinates $\left( {x{\text{ - axis}}} \right)$ which operate along the vertical wedge surface. In contrast, transverse coordinates $\left( {y{\text{ - axis}}} \right)$ are normal to the surface, as shown in Fig. 1. The calculation considers convective heat, porosity, and slip effect, where the free stream velocity is denoted as $u_{sv} = U_{sv} x^{m}$ a positive constant $U_{sv}$. The boundary wall is assumed to be in motion with a velocity slip expressed as $u = u_{v} + Ru_{y} \left( 0 \right).$ where $u_{v} > 0$ represents the stretching wedge, $u_{v} < 0$ represents the shrinking wedge, $u_{v} = 0$ represents the static wedge, and $R$ denotes the slip coefficient. Additionally, $m = {\psi \mathord{\left/ {\vphantom {\psi {\left( {2 - \psi } \right)}}} \right. \kern-0pt} {\left( {2 - \psi } \right)}},$ be the Hartree pressure gradient $\psi ,$ is the wedge angle and $\varpi = \psi \pi$ determines the wedge's total angle. When the value of $m$ is between 0 and 1, it indicates that the flow is a stagnation point flow when $\left( {\varpi = \pi } \right)$ if $m = 1$ $\left( {\psi = 1} \right)$, whereas it is a flow past a horizontal flat surface when $\left( {\varpi = 0} \right)$ for $m = 0$ $\left( {\psi = 0} \right).$ Also, $T_{f} > T_{\infty } ,$ where $T_{f}$ and $T_{\infty }$ are the surface and ambient temperatures, respectively.

The basic equations governing the steady, laminar flow of an incompressible Maxwell fluid, neglecting body forces, are

1 $$\nabla V = 0,$$

2 $$\rho_{trhnf} \, [(V\nabla ) \, V] \, = - \nabla p \, + \nabla \tau ,$$

where $V$ is the velocity vector, $\rho_{trhnf}$ is the density of the ternary hybrid nanofluid, $p$ is the pressure and $\tau$ is the extra stress tensor.

Constitutive equation for incompressible Maxwell fluid^1, 6, 8^ is:

3 $$\tau + \lambda \tau^{*} = \mu_{trhnf} A_{1} ,$$

$\lambda$ is relaxation time and overhead ‘*’ stands for the contravarient, $\mu_{trhnf}$ is the coefficient of viscosity of ternary hybrid nanofluid and $A_{1}$ is the first Rivlin-Erickson tensor given by

4 $$A_{1} = (\nabla V) + (\nabla V)^{T} ,$$

5 $$\tau^{*} = \, (V\nabla )\tau - \left[ {(\nabla V)^{T} \tau + \tau (\nabla V)} \right],$$

where superscript *T* denotes the transpose of the given matrix.

The primary assumption stated earlier forms the basis of the equations governing hybrid nanofluid flow, as presented in references^1, 6, 8^.

6 $$\frac{\partial u}{{\partial x}} + \frac{\partial v}{{\partial y}} = 0,$$

7 $$\begin{gathered} u\frac{\partial u}{{\partial x}} + v\frac{\partial u}{{\partial y}} = \frac{{\mu_{trhnf} }}{{\rho_{trhnf} }}\frac{{\partial^{2} u}}{{\partial y^{2} }} + u_{sv} \frac{{du_{sv} }}{dx} - \frac{{\mu_{trhnf} }}{{\rho_{trhnf} k^{*} }}u - \frac{F}{{\rho_{trhnf} }}u^{2} - \frac{{\lambda_{1} }}{{\rho_{trhnf} }}\left( {u^{2} \frac{{\partial^{2} u}}{{\partial x^{2} }} + v^{2} \frac{{\partial^{2} u}}{{\partial y^{2} }} + 2uv\frac{{\partial^{2} u}}{\partial xy}} \right) \hfill \\ \quad \quad \quad \quad \quad \quad \; + \frac{{\rho_{f} J_{0} M_{0} \pi }}{{8\rho_{trhnf} }}e^{{\left( {{\raise0.7ex\hbox{${ - \pi }$} \!\mathord{\left/ {\vphantom {{ - \pi } d}}\right.\kern-0pt} \!\lower0.7ex\hbox{$d$}}} \right)y}} + \frac{g}{{\rho_{trhnf} }}\left[ {\alpha_{1} \left( {T - T_{\infty } } \right) + \alpha_{2} \left( {T - T_{\infty } } \right)^{2} } \right]{\text{Cos}} \left( {{\raise0.7ex\hbox{${\pi \alpha }$} \!\mathord{\left/ {\vphantom {{\pi \alpha } 2}}\right.\kern-0pt} \!\lower0.7ex\hbox{$2$}}} \right), \hfill \\ \end{gathered}$$

8 $$u\frac{\partial T}{{\partial x}} + v\frac{\partial T}{{\partial y}} = \frac{{k_{trhnf} }}{{\left( {\rho c_{p} } \right)_{trhnf} }}\frac{{\partial^{2} T}}{{\partial y^{2} }} + \frac{{\mu_{trhnf} }}{{\left( {\rho c_{p} } \right)_{trhnf} }}\left( {\frac{\partial u}{{\partial y}}} \right)^{2} + \frac{{Q_{0} }}{{\left( {\rho c_{p} } \right)_{trhnf} }}\left( {T - T_{\infty } } \right) - \frac{1}{{\left( {\rho c_{p} } \right)_{trhnf} }}\frac{{\partial q_{r} }}{\partial y},$$

Boundary conditions are:

9 $$\left. \begin{gathered} v\left( {x,\,0} \right) = 0,\,\,\,\,u\left( {x,\,0} \right) = u_{v} + \mu_{trhnf} \frac{\partial u}{{\partial y}},\,\,\,\, - k_{trhnf} \frac{\partial T}{{\partial y}} = h_{f} \left( {T - T_{w} } \right),\,\,\,\,\,{\text{at}}\,\,y \to 0, \hfill \\ u \to u_{sv} ,\,\,\,T \to T_{\infty } ,\,\,\,{\text{at}}\,\,y \to \infty , \hfill \\ \end{gathered} \right\}$$

In this context, it represents the velocity *v* in the *x* direction, and *u* denotes the velocity in the *y* direction. The current density of the electrodes is represented by $$J_{0}$$, the heat transfer coefficient is $$h_{f} ,$$ and the permanent magnet is referred to as $$M_{0} .$$ Table 1 summarizes the thermal properties of nanofluids, hybrid nanofluids, and ternary nanofluids. The table includes the volume percentages of $${\text{Al}}_{{2}} {\text{O}}_{{3}} ,$$ Cu and $${\text{TiO}}_{2}$$ nanomaterials, which are $$\phi_{1} ,$$ $$\phi_{2}$$ and $$\phi_{3}$$, respectively. To convert the nanofluids, hybrid nanofluids, and ternary nanofluids to a Maxwell fluid putting $$\phi_{1} = \phi_{2} = \phi_{3} = 0$$, the equation is utilized, which represents the heat capacity $$\left( {\rho C} \right)p_{trhnf} ,$$ electrical conductivity $$\sigma_{trhnf} ,$$ the liquid density $$\rho_{trhnf} ,$$ specific heat $$Cp_{trhnf} ,$$ dynamic viscosity $$\mu_{trhnf}$$ and thermal conductivity of ternary hybrid nanofluid $$k_{trhnf} .$$ The subscripts $$f,$$ $$nf,$$ $$hnf,$$ $$trhnf,$$ $$s_{1} ,$$$$s_{2}$$ and $$s_{3}$$ indicate the fluid, nanofluid, hybrid nanofluid, ternary hybrid nanofluid, and solid components of $${\text{Al}}_{{2}} {\text{O}}_{{3}} ,$$ Cu and $${\text{TiO}}_{2}$$ nanoparticles. Additionally, Eq. (14) outlines the physical properties of the engine oil $${\text{Al}}_{{2}} {\text{O}}_{{3}}$$, Cu, and $${\text{TiO}}_{2}$$ nanoparticles.

**Table 1:** The ${\text{Al}}_{{2}} {\text{O}}_{{3}}$ and ${\text{Cu}}$ thermo-physical properties along with ${\text{TiO}}_{2}$ and E.O^31–36^.

Physical properties	$\rho \left( {{\text{kg}}/{\text{m}}^{3} } \right)$	$\rho c_{p} \left( {{\text{J}}/{\text{kgK}}} \right)$	$k\left( {W/{\text{mK}}} \right)$
EO	884	1910	0.144
${\text{Al}}_{{2}} {\text{O}}_{{3}}$	3970	765	40
${\text{Cu}}$	8933	385	401
${\text{TiO}}_{2}$	4230	692	8.4

Equations (6)–(8) and the constraints (9) are subject to similarity transformations, as shown in Eq. (10) in reference^8^, where the stream function $\left( \omega \right)$ is given by $u = {{\partial \omega } \mathord{\left/ {\vphantom {{\partial \omega } {\partial y,}}} \right. \kern-0pt} {\partial y,}}$ $\,{\text{and}}\,\,v = - {{\partial \omega } \mathord{\left/ {\vphantom {{\partial \omega } {\partial x\,\,\,}}} \right. \kern-0pt} {\partial x\,\,\,}}$ while $\eta$ be a similarity variable.

10 $$\left. \begin{gathered} \omega = \sqrt {U_{sv} \nu_{f} } x^{{{\raise0.7ex\hbox{${\left( {m + 1} \right)}$} \!\mathord{\left/ {\vphantom {{\left( {m + 1} \right)} 2}}\right.\kern-0pt} \!\lower0.7ex\hbox{$2$}}}} f\left( \eta \right),\,\,\,\,\eta = \sqrt {\frac{{U_{sv} }}{{\nu_{f} }}} x^{{{\raise0.7ex\hbox{${\left( {m - 1} \right)}$} \!\mathord{\left/ {\vphantom {{\left( {m - 1} \right)} 2}}\right.\kern-0pt} \!\lower0.7ex\hbox{$2$}}}} y,\,\,\,\theta \left( \eta \right) = \frac{{T - T_{\infty } }}{{T_{f} - T_{\infty } }},\,\,u = U_{sv} x^{m} f^{\prime}\left( \eta \right), \hfill \\ v = - \sqrt {U_{sv} \nu_{f} } x^{{{\raise0.7ex\hbox{${\left( {m - 1} \right)}$} \!\mathord{\left/ {\vphantom {{\left( {m - 1} \right)} 2}}\right.\kern-0pt} \!\lower0.7ex\hbox{$2$}}}} \left( {\frac{m + 1}{2}} \right)\left( {\frac{m - 1}{{m + 1}}\eta f^{\prime}\left( \eta \right) + f\left( \eta \right)} \right). \hfill \\ \end{gathered} \right\}$$

Equations (7)–(9) transformed into a collection of nonlinear ODEs by employing similarity transformations (10):

11 $$\begin{gathered} \frac{{\mu_{r} }}{{\rho_{r} }}\left( {\frac{m + 1}{2}} \right)f^{\prime\prime\prime} - \left( {f^{\prime}} \right)^{2} + \left( {\frac{m + 1}{2}} \right)ff^{\prime\prime} + m + \frac{{{\text{Mh}}\exp \left( { - ah\eta } \right)}}{{\rho_{r} }} - \frac{{\mu_{r} }}{{\rho_{r} }}K_{p} f^{\prime} - \frac{{F_{r} }}{{\rho_{r} }}\left( {f^{\prime}} \right)^{2} \hfill \\ \quad - \frac{\lambda }{{\rho_{r} }}\left( \begin{gathered} m\left( {m - 1} \right)\left( {f^{\prime}} \right)^{3} + \left( {\frac{m - 1}{2}} \right)^{2} \eta \left( {f^{\prime}} \right)^{2} f^{\prime\prime} + \left( {\frac{m + 1}{2}} \right)^{2} f^{2} f^{\prime\prime\prime} \hfill \\ - \left( {\frac{m - 1}{2}} \right)^{2} \eta^{2} f^{\prime\prime\prime}\left( {f^{\prime}} \right) + m\left( {\frac{m + 1}{2}} \right)ff^{\prime}f^{\prime\prime} + \left( {\frac{{m^{2} - 1}}{4}} \right)ff^{\prime}f^{\prime\prime} \hfill \\ \end{gathered} \right) + \frac{{\beta_{1} }}{{\rho_{r} }}\theta \left( {1 + \beta_{2} \theta } \right) = 0, \hfill \\ \end{gathered}$$

12 $$\begin{gathered} \frac{{k_{r} }}{{\left( {\rho Cp} \right)_{r} }}\theta^{\prime\prime} + \frac{Nr}{{\left( {\rho Cp} \right)_{r} }}\left( {1 + \theta \left( {\theta_{r} - 1} \right)} \right)^{3} \theta^{\prime\prime} + \frac{3Nr}{{\left( {\rho Cp} \right)_{r} }}\left( {\theta^{\prime}} \right)^{2} \left( {\theta_{r} - 1} \right)\left( {1 + \theta \left( {\theta_{r} - 1} \right)} \right)^{2} \hfill \\ \quad + \frac{2\Pr \theta H}{{\left( {\rho Cp} \right)_{r} \left( {m + 1} \right)}} + \Pr f\theta^{\prime} + \frac{{\Pr Ec\mu_{r} }}{{\left( {\rho Cp} \right)_{r} }}\left( {f^{\prime\prime}} \right)^{2} = 0, \hfill \\ \end{gathered}$$

with boundary conditions are

13 $$\left. \begin{gathered} f\left( \eta \right) = 0,\,\,f^{\prime}\left( \eta \right) = 1 + \lambda \mu_{r} f^{\prime\prime}\left( \eta \right),\,\,k_{r} \theta^{\prime}\left( \eta \right) = - Bi\left( {1 - \theta \left( \eta \right)} \right),\,\,\,\,{\text{at}}\,\,\,\eta \to 0, \hfill \\ f^{\prime}\left( \eta \right) = 1,\,\,f^{\prime\prime}\left( \eta \right) = 0,\,\,\theta \left( \eta \right) = 0,\,\,{\text{at}}\,\,\,\eta \to \infty , \hfill \\ \end{gathered} \right\}$$

where Modified Hartmann number ${\text{Mh}} = {{\pi j_{0} M_{0} } \mathord{\left/ {\vphantom {{\pi j_{0} M_{0} } {4u_{sv}^{2} }}} \right. \kern-0pt} {4u_{sv}^{2} }},$ second-grade fluid parameter $\lambda = {{2\lambda_{1} b} \mathord{\left/ {\vphantom {{2\lambda_{1} b} {\mu_{f} }}} \right. \kern-0pt} {\mu_{f} }},$ non-dimensional parameter $ah = \frac{\pi }{d}\sqrt {{{2\nu_{f} } \mathord{\left/ {\vphantom {{2\nu_{f} } {m + 1}}} \right. \kern-0pt} {m + 1}}} ,$ slip parameter $\lambda = R\mu_{f} \sqrt {{{U_{sv} } \mathord{\left/ {\vphantom {{U_{sv} } {v_{f} }}} \right. \kern-0pt} {v_{f} }}} ,$ Prandtl number $\Pr = {{\alpha_{f} } \mathord{\left/ {\vphantom {{\alpha_{f} } {v_{f} }}} \right. \kern-0pt} {v_{f} }},$ heat source parameter $H = {{Q_{0} } \mathord{\left/ {\vphantom {{Q_{0} } {\left( {\rho Cp} \right)_{f} }}} \right. \kern-0pt} {\left( {\rho Cp} \right)_{f} }},$ thermal radiation ${\text{Nr}} = {{16\delta^{*} T_{\infty }^{3} a} \mathord{\left/ {\vphantom {{16\delta^{*} T_{\infty }^{3} a} {3k^{*} k_{f} }}} \right. \kern-0pt} {3k^{*} k_{f} }},$ Biot number $Bi = \frac{{h_{f} }}{{k_{f} }}\sqrt {{{2xv_{f} } \mathord{\left/ {\vphantom {{2xv_{f} } {U_{sv} \left( {m + 1} \right)}}} \right. \kern-0pt} {U_{sv} \left( {m + 1} \right)}}} ,$ convection parameter $\beta_{1} = {{Grx} \mathord{\left/ {\vphantom {{Grx} {{\text{Re}}_{x}^{2} }}} \right. \kern-0pt} {{\text{Re}}_{x}^{2} }},$ nonlinear convection parameter for temperature $\beta_{2} = {{\alpha_{2} \left( {T_{w} - T_{\infty } } \right)} \mathord{\left/ {\vphantom {{\alpha_{2} \left( {T_{w} - T_{\infty } } \right)} {\alpha_{1} }}} \right. \kern-0pt} {\alpha_{1} }},$ and temperature difference $\theta_{r} = {{T_{f} } \mathord{\left/ {\vphantom {{T_{f} } {T_{\infty } }}} \right. \kern-0pt} {T_{\infty } }}.$

The thermophysical characteristics of the hybrid nanofluids have been documented in references^36–42, 45^:

14 $$\left. \begin{gathered} \rho_{r} = \frac{{\rho_{trhnf} }}{{\rho_{f} }} = \left( {1 - \phi_{3} } \right)\left[ {\left( {1 - \phi_{2} } \right)\left\{ {\left( {1 - \phi_{1} } \right) + \frac{{\rho_{{s_{1} }} \phi_{1} }}{{\rho_{f} }}} \right\} + \frac{{\rho_{{s_{2} }} \phi_{2} }}{{\rho_{f} }}} \right] + \frac{{\rho_{{s_{3} }} \phi_{3} }}{{\rho_{f} }}, \hfill \\ \mu_{r} = \frac{{\mu_{trhnf} }}{{\mu_{f} }} = \left( {1 - \phi_{1} } \right)^{ - 2.5} \left( {1 - \phi_{2} } \right)^{ - 2.5} \left( {1 - \phi_{3} } \right)^{ - 2.5} , \hfill \\ \left( {\rho C_{\rho } } \right)_{r} = \frac{{\left( {\rho C_{\rho } } \right)_{trhnf} }}{{\left( {\rho C_{\rho } } \right)_{f} }} = \frac{{\phi_{3} \left( {\rho C_{\rho } } \right)_{{s_{3} }} }}{{\left( {\rho C_{\rho } } \right)_{f} }} + \left( {1 - \phi_{3} } \right)\left[ {\left( {1 - \phi_{2} } \right)\left\{ {\left( {1 - \phi_{1} } \right) + \frac{{\left( {\rho C_{\rho } } \right)_{{s_{1} }} \phi_{1} }}{{\left( {\rho C_{\rho } } \right)_{f} }}} \right\} + \frac{{\left( {\rho C_{\rho } } \right)_{{s_{2} }} \phi_{2} }}{{\left( {\rho C_{\rho } } \right)_{f} }}} \right], \hfill \\ k_{r} = \frac{{k_{trhnf} }}{{k_{hnf} }} = \frac{{2k_{nf} - 2\phi_{1} \left( {k_{{s_{1} }} - k_{nf} } \right) + k_{{s_{1} }} }}{{2k_{nf} + \phi_{1} \left( {k_{{s_{1} }} - k_{nf} } \right) + k_{{s_{1} }} }},\frac{{k_{hnf} }}{{k_{nf} }} = \frac{{2k_{f} - 2\phi_{2} \left( {k_{{s_{2} }} - k_{f} } \right) + k_{{s_{2} }} }}{{2k_{f} + \phi_{2} \left( {k_{{s_{2} }} - k_{f} } \right) + k_{{s_{2} }} }}, \hfill \\ \frac{{k_{nf} }}{{k_{f} }} = \frac{{2k_{f} - 2\phi_{3} \left( {k_{{s_{3} }} - k_{f} } \right) + k_{{s_{3} }} }}{{2k_{f} + \phi_{3} \left( {k_{{s_{3} }} - k_{f} } \right) + k_{{s_{3} }} }}. \hfill \\ \end{gathered} \right\}$$

### Physical interest

The skin friction $\left( {Cf_{x} } \right)$ and Nusslt number $\left( {{\text{Nu}}_{x} } \right)$ are defined by^5, 8^

15 $$Cf_{x} = \frac{1}{{\rho_{f} u_{e}^{2} }}\left[ {\mu_{hnf} \left( {1 + \lambda_{1} } \right)\frac{\partial u}{{\partial y}}} \right]_{y = 0} .$$

16 $${\text{Nu}}_{x} = - \frac{x}{{k_{f} \left( {T_{w} - T_{\infty } } \right)}}\left[ {k_{hnf} + \frac{{16\sigma^{*} T_{\infty }^{3} }}{{3k^{*} }}} \right]\left( {\frac{\partial T}{{\partial y}}} \right)_{y = 0} .$$

Then incorporate (10) to (15) and (16), resulting in the relationship shown below:

17 $$\sqrt {{\text{Re}}_{x} } C_{fx} = \mu_{r} \left( {1 + \lambda } \right)f^{\prime\prime}\left( 0 \right),$$

18 $$\left( {{\text{Re}}_{x} } \right)^{ - 0.5} Nu_{x} = - \left( {k_{r} + Nr\left( {1 + \theta \left( 0 \right)\left( {\theta_{w} - 1} \right)} \right)^{3} } \right)\theta^{\prime}\left( 0 \right),$$

${\text{Re}}_{x} = {{u_{e} x} \mathord{\left/ {\vphantom {{u_{e} x} {\nu_{f} }}} \right. \kern-0pt} {\nu_{f} }}$ be the local Reynolds number along the *x*-axis.

### Fuzzification

A slight alteration in nanoparticle volume fraction can control the temperature profile of nanofluids and hybrid nanofluids in practical situations. The nanoparticle volume fraction is approached as a fuzzy number to tackle this problem using the TFN method (see Table 2). The governing ODEs are then converted into FDEs using the $\sigma {\text{ - cut}}$. The parameter varies between 0 and 1, and it determines the degree of fuzziness of the TFN.

**Table 2:** In terms of TFN, the following parameters are detailed^45–47^.

Fuzzy Numbers	Crisp value	TFN	$\sigma {\text{ - cut}}$ approach
$$\phi_{1}$$$$\left( {{\text{Al}}_{{2}} {\text{O}}_{{3}} } \right)$$	[0.01–0.04]	[0, 0.05, 0.1]	$$\left[ {0.05\sigma ,\,\,0.1 - 0.05\sigma } \right],\,\,\sigma \in \left[ {0,\,1} \right]$$
$$\phi_{2}$$$$\left( {{\text{Cu}}} \right)$$	[0.01–0.04]	[0, 0.05, 0.1]	$$\left[ {0.05\sigma ,\,\,0.1 - 0.05\sigma } \right],\,\,\sigma \in \left[ {0,\,1} \right]$$
$\phi_{3}$ $\left( {{\text{TiO}}_{2} } \right)$	[0.01–0.04]	[0, 0.05, 0.1]	$\left[ {0.05\sigma ,\,\,0.1 - 0.05\sigma } \right],\,\,\sigma \in \left[ {0,\,1} \right]$

**Figure 2:**
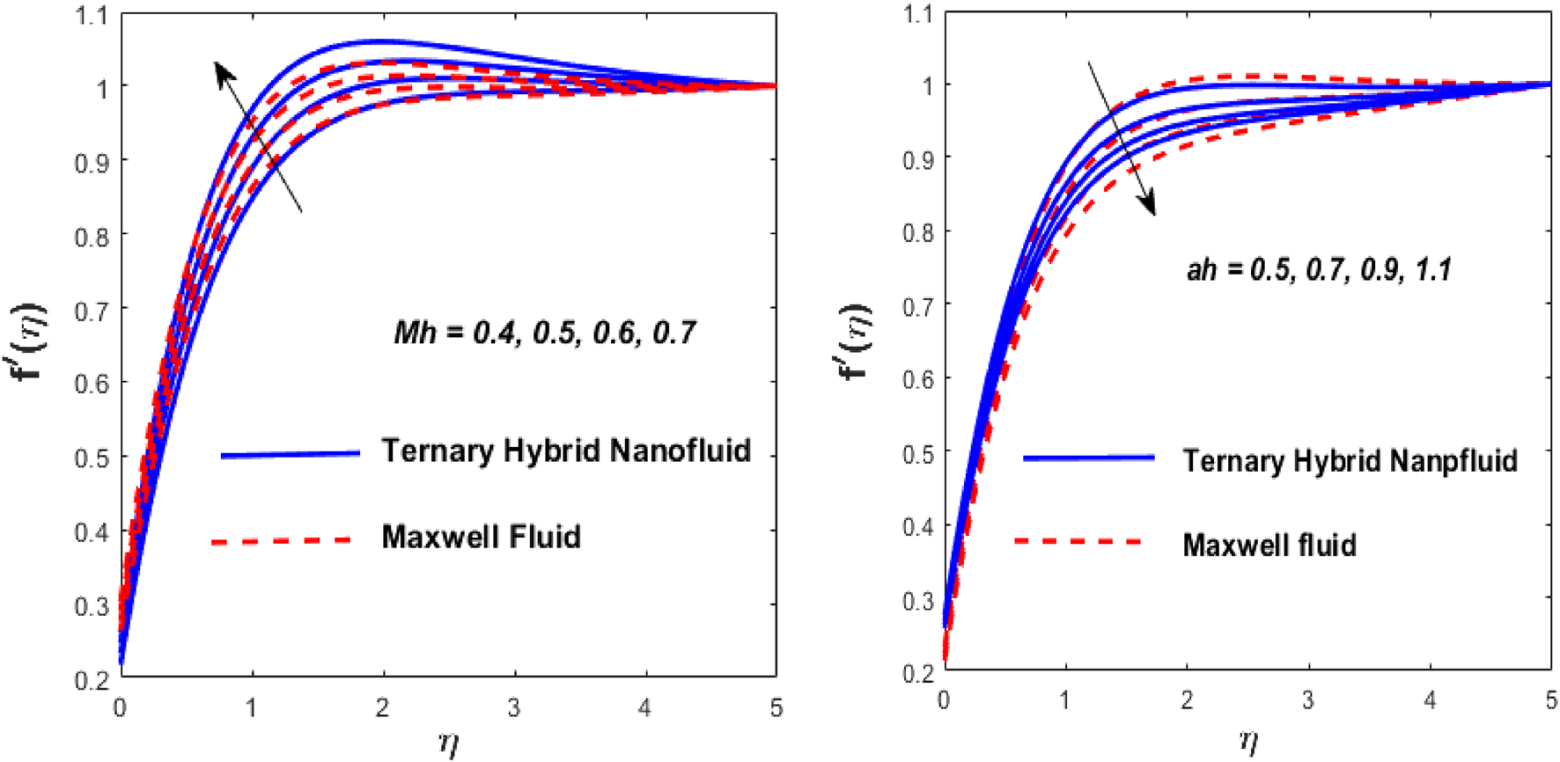
Impact of ${\text{Mh}}$ and $ah$ on $f^{\prime}\left( \eta \right).$

Let $\phi_{1} = \phi_{2} = \phi_{3} = \left[ { \, 0,0.05,0.1} \right]$ be a TFN, defined by three parameters: 0 (lower bound), 0.05 (most likely value), and 0.1 (upper bound), which is represented in Fig. 2. In Eq. (19), the membership function of the TFN can be expressed using TFN.

19 $${\text{Membership}}\;{\text{function}} = \left\{ \begin{gathered} \frac{0 - \eta }{{0.05 - 0}}\quad \quad {\text{for}}\;\eta \in [0,\,\,0.05], \hfill \\ \frac{\eta - 0.1}{{0.1 - 0.05}}\quad {\text{for}}\;\eta \in [0.05,\,\,0.1], \hfill \\ 0,\quad \quad \quad \quad \;\;{\text{otherwise}}. \hfill \\ \end{gathered} \right.$$

The approach for converting TFNs into interval numbers $\sigma {\text{ - cut}}$ is expressed as $\overline{\theta }\left( {\eta ,\,\sigma } \right) = \left[ {\theta_{1} (\eta ,\,\,\sigma ),\theta_{2} (\eta ,\,\,\sigma )} \right] = \left[ {0 + \sigma (0.05 - 0),\,\,0.1 - \sigma (0.1 - 0.05)} \right],$ being the key factor in the transformation process, where $0 \le \sigma {\text{ - cut}} \le 1.$

Use the FDEs via $\sigma {\text{ - cut}}$ technique to handle such TFNs. The FDEs are renewed into lower $\theta_{1} (\eta ,\,\,\sigma )$ and upper bounds $\theta_{2} (\eta ,\,\,\sigma ).$ Additional information on this topic can be found in the literature^43–46^.

### Numerical scheme

The governing flow equations of the fluid model exhibit significant nonlinearity, making it impractical to obtain exact solutions due to their high complexity. Therefore, the built-in Matlab software bvp4c numerical method is used to obtain a solution for such a problem. Also, bvp4c is a finite difference code that implements the three-stage Lobatto IIIa formula. One such method involves transforming the governing problem into a set of associated first-order equations, which is the subject of the current discussion. This is a collocation formula, and the collocation polynomial provides a C1-continuous solution that is fourth-order accurate uniformly in the interval of integration^24^. Here, the step size 0.001 is chosen to get the desired convergence criterion of $10^{ - 6}$ the problem. This subsection presents the nonlinearly generated ODEs (11)–(12) and the boundary constraints (13).

20 $$f\left( \eta \right) = G_{1} ,\,f^{\prime}\left( \eta \right) = G_{2} ,\,\,f^{\prime\prime}\left( \eta \right) = G_{3} ,\,\,f^{\prime\prime\prime}\left( \eta \right) = G_{4} ,\,\,f^{\prime\prime\prime\prime}\left( \eta \right) = G^{\prime}_{4} ,\,\,\theta \left( \eta \right) = G_{5} ,\,\,\theta ^{\prime}\left( \eta \right) = G_{6} ,$$

21 $$G^{\prime}_{4} = \frac{{2\rho_{r} }}{{\alpha \left( {m + 1} \right)G_{1} }}\left[ \begin{gathered} \frac{{\mu_{r} }}{{\rho_{r} }}G_{4} + \left( {\frac{2m}{{m + 1}}} \right)\left( {1 - \left( {G_{2} } \right)^{2} } \right) + \frac{\lambda }{{\rho_{r} }}\left( \begin{gathered} \left( {3m - 1} \right)G_{2} G_{3} + \left( {\frac{3m - 1}{2}} \right)G_{3}^{2} \hfill \\ + \left( {m - 1} \right)\eta G_{3} G_{4} \hfill \\ \end{gathered} \right) \hfill \\ + {\text{Mh}}\exp \left( { - ah\eta } \right) + G_{1} G_{3} \hfill \\ \end{gathered} \right],$$

22 $$G^{\prime}_{6} = = \frac{{ - \left( {\rho Cp} \right)_{r} }}{{k_{r} + \left( {1 + G_{5} \left( {\theta_{r} - 1} \right)} \right)^{3} }}\left[ \begin{gathered} \frac{3Nr}{{\left( {\rho Cp} \right)_{r} }}\left( {G_{6} } \right)^{2} \left( {\theta_{r} - 1} \right)\left( {1 + G_{5} \left( {\theta_{r} - 1} \right)} \right)^{2} + \frac{{\Pr G_{5} H}}{{\left( {\rho Cp} \right)_{r} }} \hfill \\ + \Pr G_{1} G_{6} \hfill \\ \end{gathered} \right],$$

with boundary conditions are

23 $$\left. \begin{gathered} G_{1} = 0,\,\,G_{2} = S + \lambda \mu_{r} G_{3} \left( \eta \right),\,\,k_{r} G_{6} \left( \eta \right) = - Bi\left( {1 - G_{1} \left( \eta \right)} \right),\,\,\,\,{\text{at}}\,\,\,\eta \to 0, \hfill \\ G_{2} \left( \eta \right) = 1,\,\,G_{3} \left( \eta \right) = 0,\,\,G_{5} \left( \eta \right) = 0,\,\,{\text{at}}\,\,\,\eta \to \infty . \hfill \\ \end{gathered} \right\}$$

By utilizing the bvp4c algorithm in MATLAB, the set mentioned above of ODEs (20) and (22), along with the B.C.s (23), can be numerically solved.

## Results and discussion

This research aims to use maxwell ternary hybrid nanofluids flowing over a Riga wedge for efficient cooling and heating applications in thermal engineering. The ternary hybrid nanofluid comprises solid nanoparticles such as ${\text{Al}}_{{2}} {\text{O}}_{{3}} {,}$ Cu and ${\text{TiO}}_{{2}}$ with E.O. as a base liquid.

**Figure 3:**
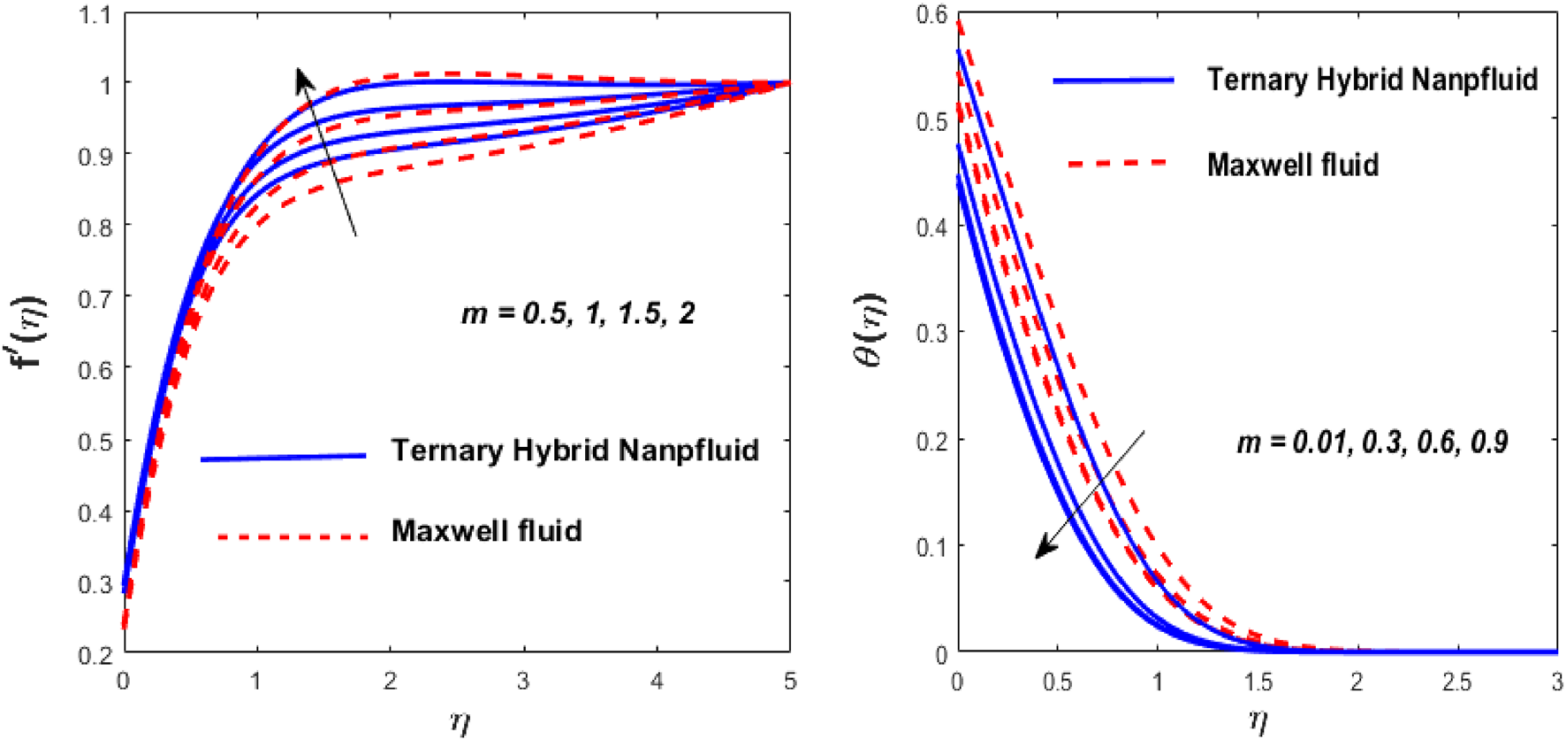
Impact of $m$ on $f^{\prime}\left( \eta \right)$ and $\theta \left( \eta \right).$

**Figure 4:**
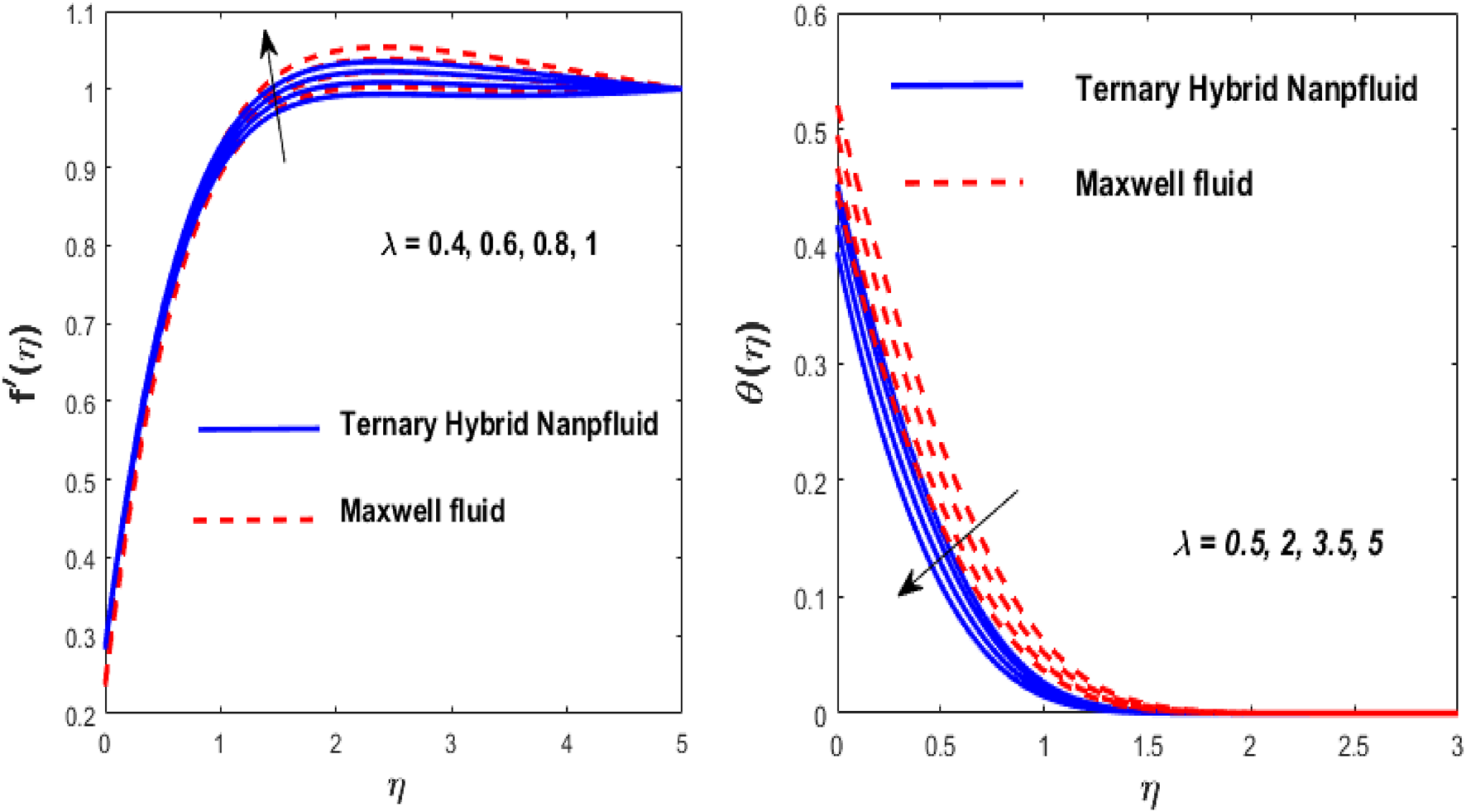
Impact of $\lambda$ on $f^{\prime}\left( \eta \right)$ and $\theta \left( \eta \right).$

**Figure 5:**
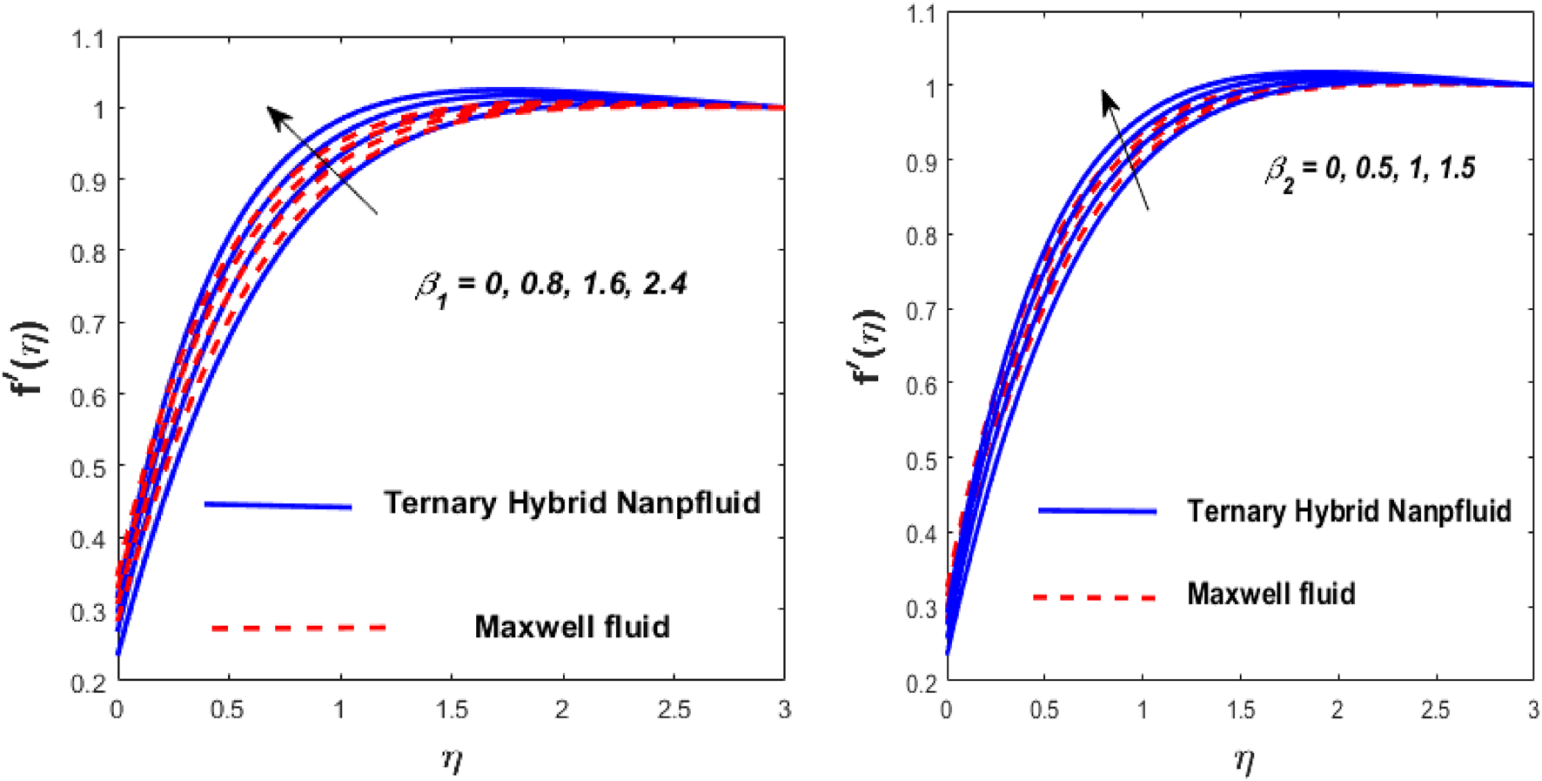
Impact of $\beta_{1}$ and $\beta_{2}$ on $f^{\prime}\left( \eta \right).$

**Figure 6:**
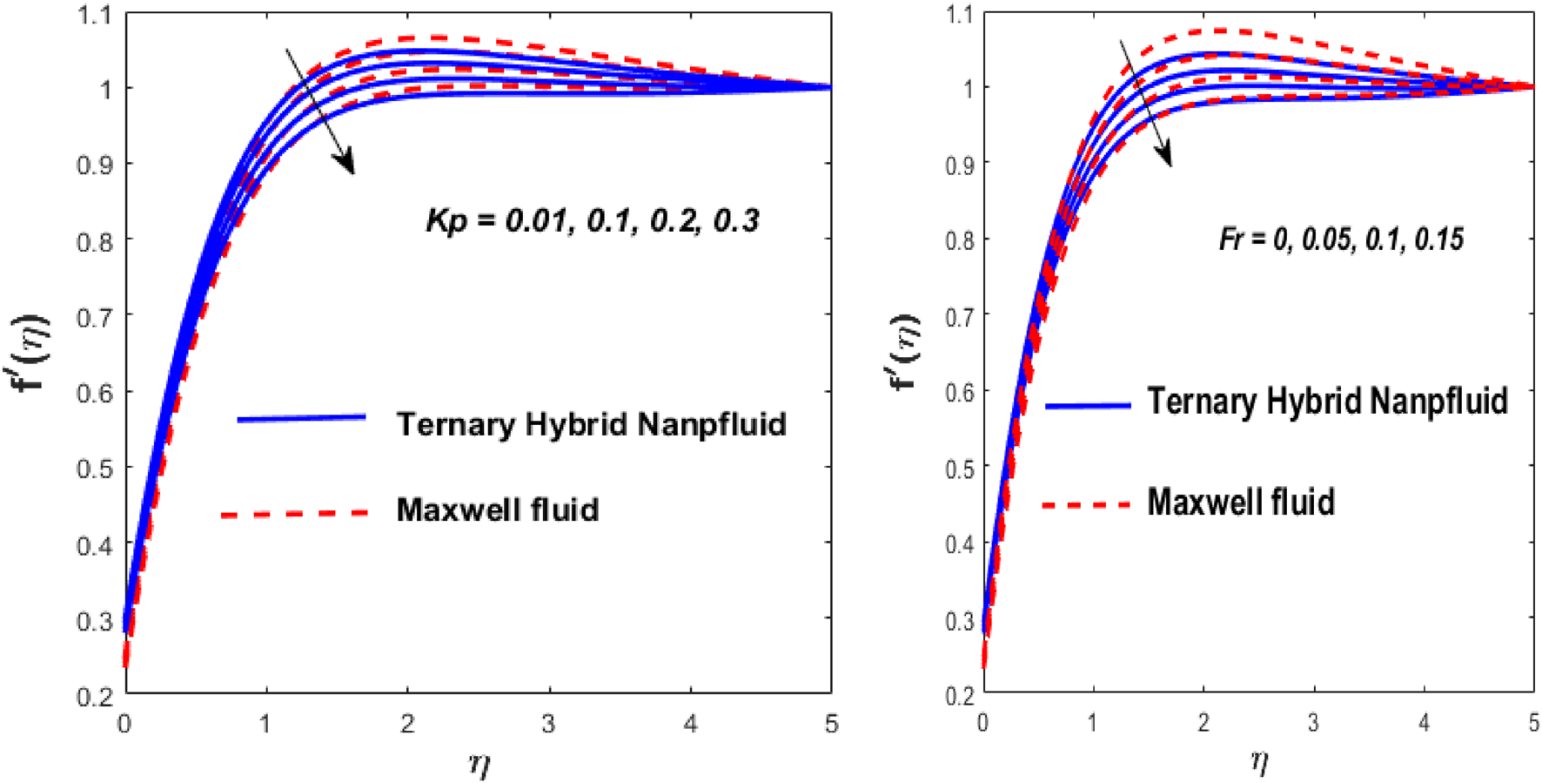
Impact of $Kp$ and $Fr$ on $f^{\prime}\left( \eta \right).$

**Figure 7:**
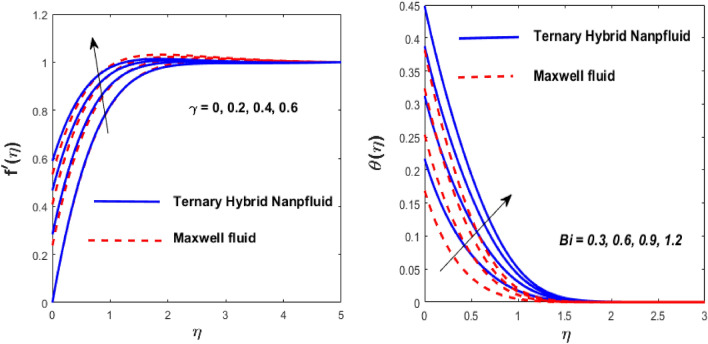
Impact of $\gamma$ and $Bi$ on $f^{\prime}\left( \eta \right)$ and $\theta \left( \eta \right).$

**Figure 8:**
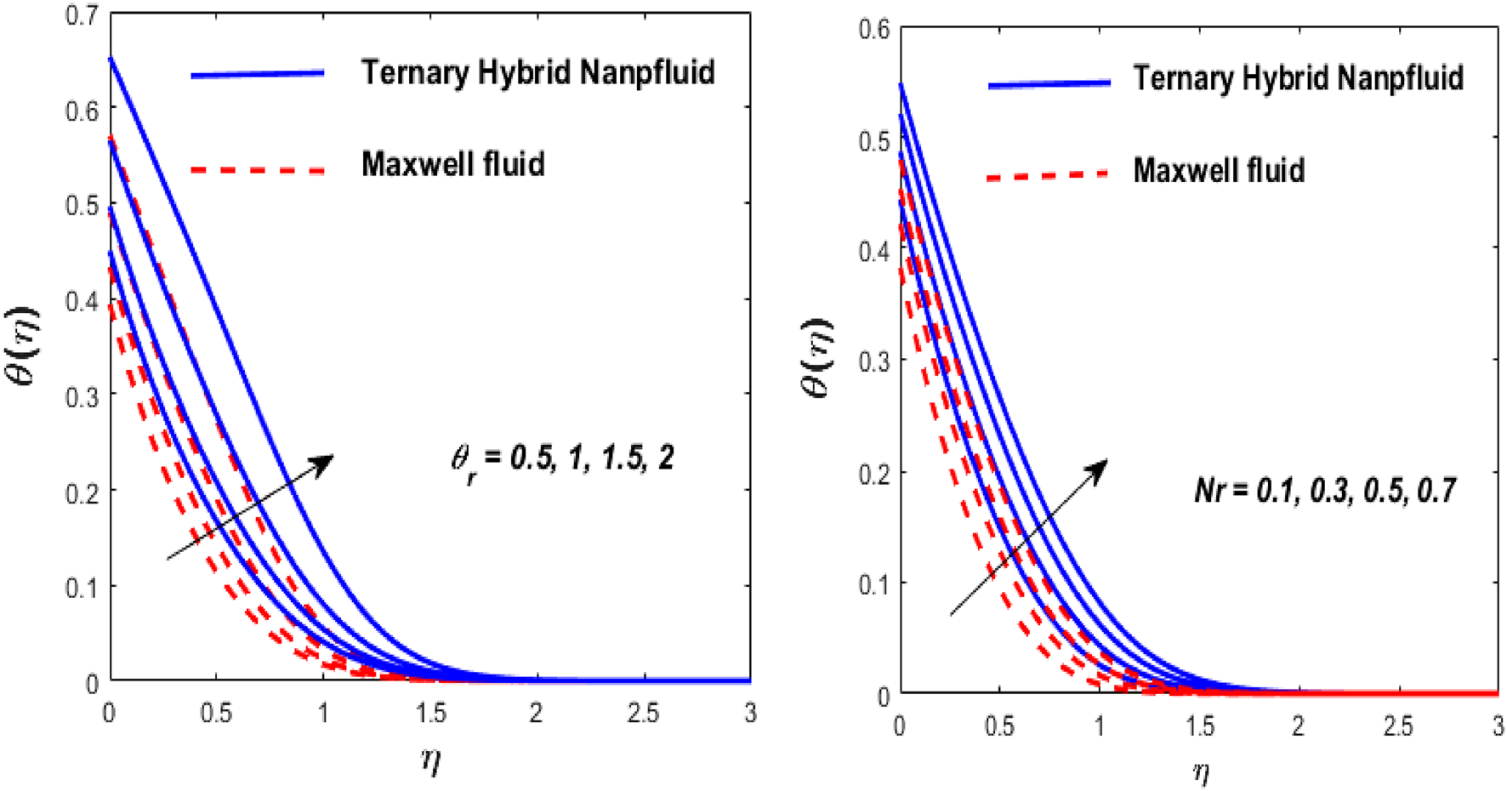
Impact of $\theta_{r}$ and ${\text{Nr}}$ on $\theta \left( \eta \right).$

**Figure 9:**
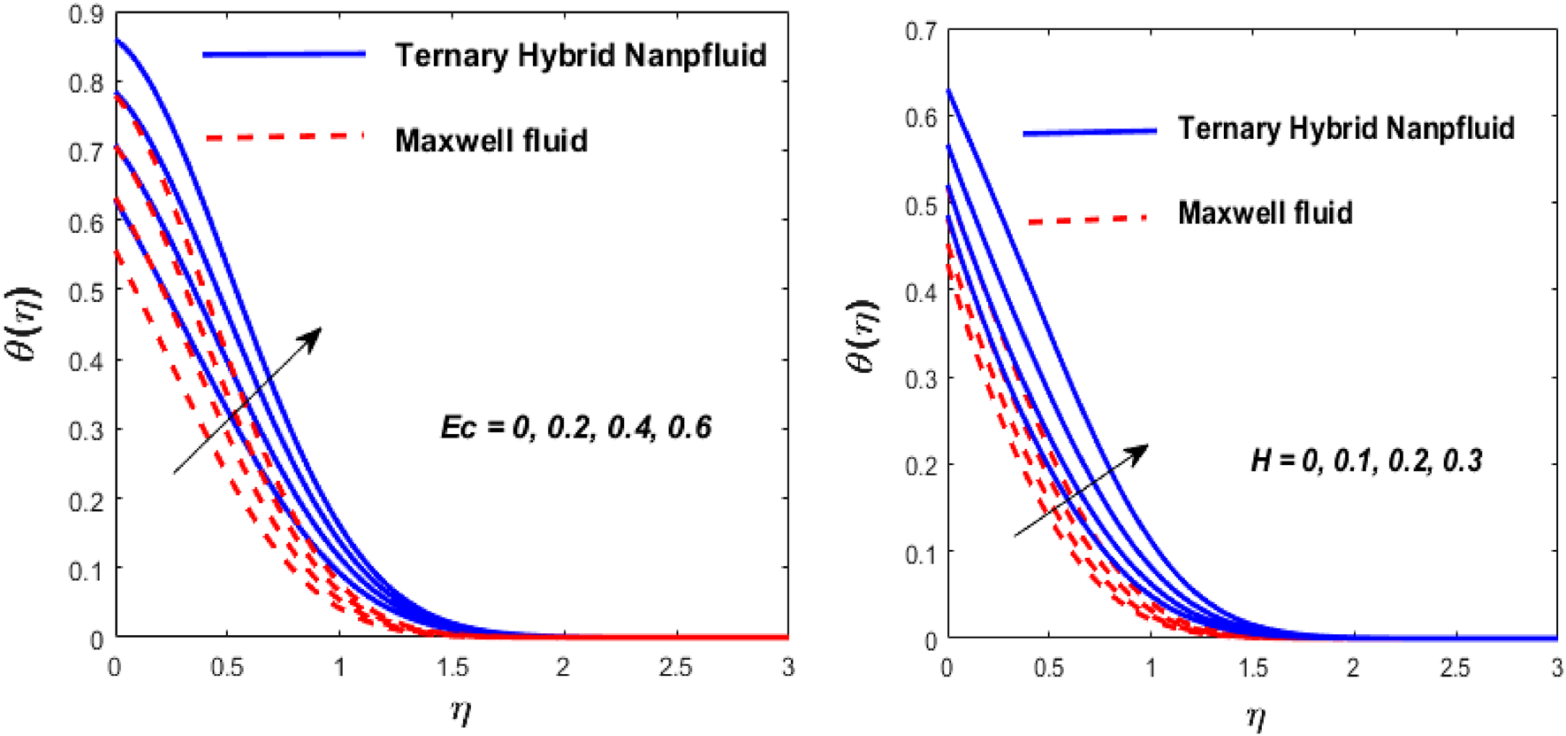
Impact of $Ec$ and $H$ on $\theta \left( \eta \right).$

**Figure 10:**
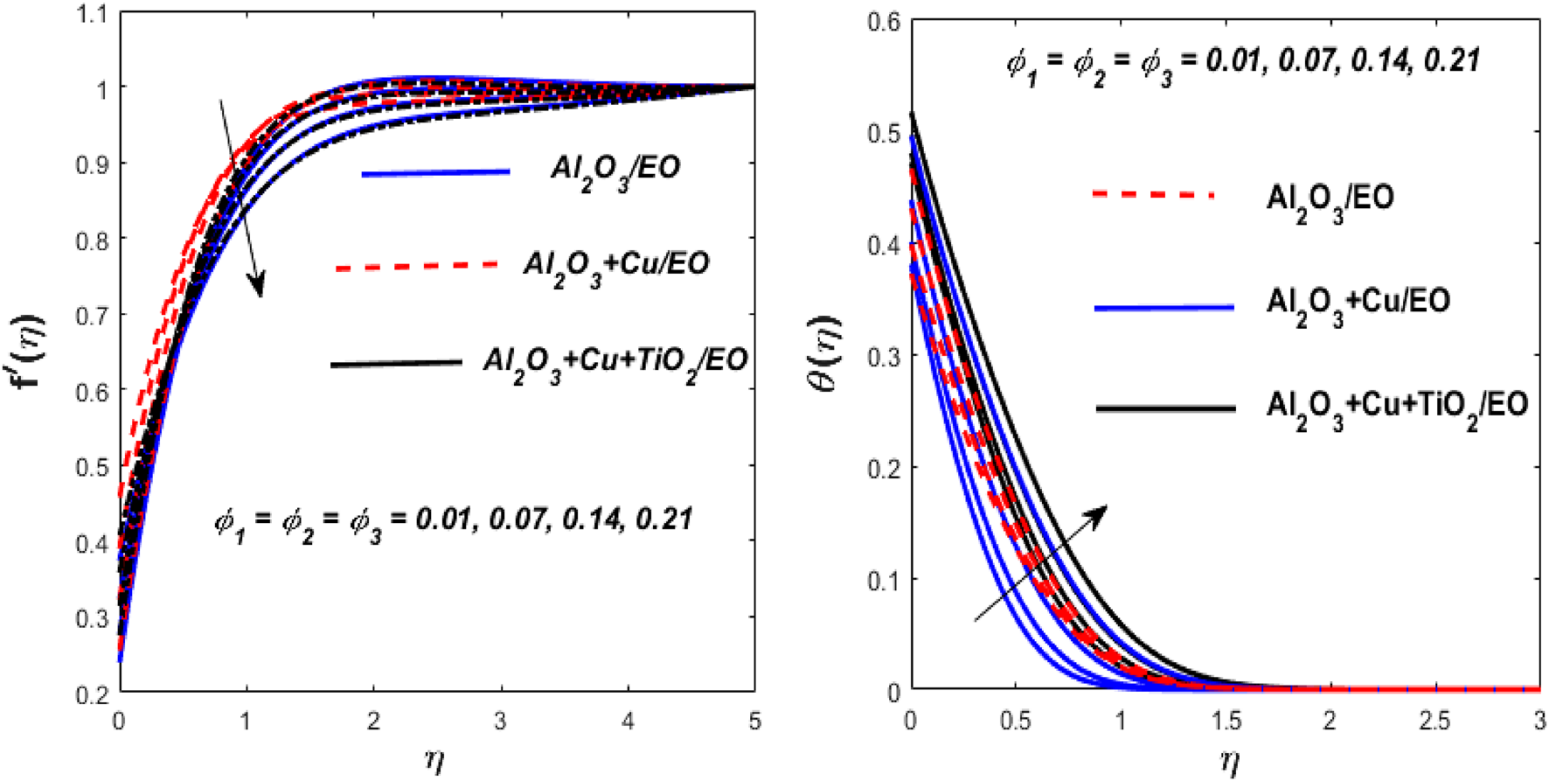
Impact of $\phi_{1} ,$ $\phi_{2}$ and $\phi_{3}$ on $f^{\prime}\left( \eta \right)$ and $\theta \left( \eta \right).$

**Figure 11:**
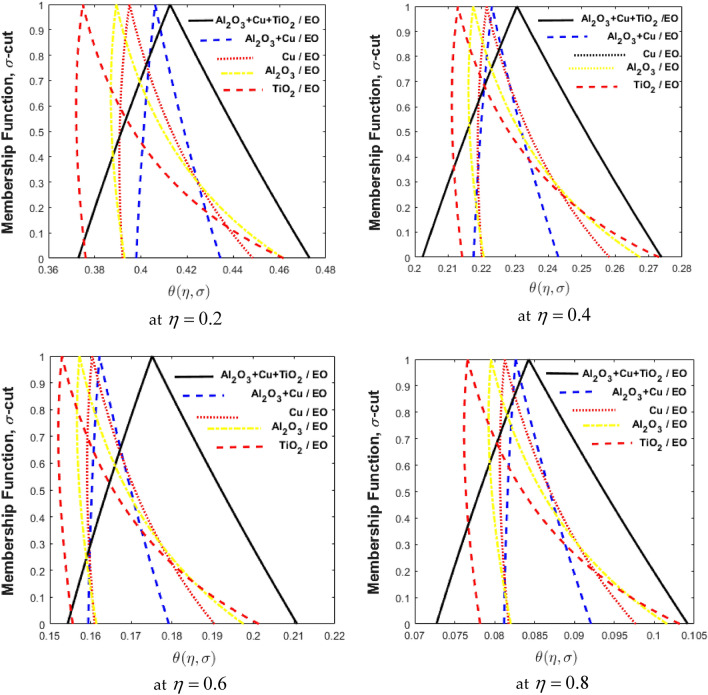
Comparison of nanofluids, hybrid nanofluids, and ternary hybrid nanofluids for varying of η.

The solid particles dissolve in the base liquid, resulting in the formation of the tri-hybrid nanofluid. Thermal properties were found using the combination ${\text{Al}}_{{2}} {\text{O}}_{{3}} + {\text{Cu}} + {\text{TiO}}_{{2}} {\text{/EO}}$ of the effect of nonlinear thermal radiations and convection associated with the porous surface. The impact of the constraints is depicted in Figs. 2, 3, 4, 5, 6, 7, 8, 9, 10 and 11.

Table 3 compares drag force and heat transmission rate with existing data from Kumari et al.^51^ and Rajput et al.^52^ for numerous *m* values to ensure the above scheme's accuracy. An exceptional match was witnessed, which validates the numerical technique and the resultant outcomes.

**Table 3:** Comparison of $$- f^{\prime\prime}\left( 0 \right)$$ and $$- \theta^{\prime}\left( 0 \right)$$ for several values of *m* when $$Kp = H = Mh =$$ Nr = $$\lambda =$$$$\beta_{1} =$$$$Nr = Ec = 0,\,\,\Pr = 0.73.$$

*m*	Kumari et al.^51^		Rajput et al.^52^		Present results	
	$- f^{\prime\prime}\left( 0 \right)$	$- \theta^{\prime}\left( 0 \right)$	$- f^{\prime\prime}\left( 0 \right)$	$- \theta^{\prime}\left( 0 \right)$	$- f^{\prime\prime}\left( 0 \right)$	$- \theta^{\prime}\left( 0 \right)$
0	0.46975	0.42079	0.46960	0.42015	0.46960	0.42014
0.0909	0.65501	0.44770	0.65498	0.44729	0.65487	0.44728
0.2	0.80214	0.46534	0.80213	0.46503	0.80212	0.46501
0.3333	0.92766	0.47840	0.92765	0.47814	0.92745	0.47815

The inspiration of the modified Hartmann number (Mh) and the dimensionless parameter (*ah*) on $f^{\prime}\left( \eta \right)$ is appreciated in Fig. 2. The argument is that for both fluids, velocity improves as Mh rises and falls as *ah* grows. The complex principles of the Mh elevate the magnetism between the wedge's boundary. Due to increased magnetism between the wedge boundary, the Riga wedge is more compact than the ordinary wedge. Also, Mh is associated with the Riga layout, which reduces and accelerates the stream's friction. The wedge parameter (*m*) outcomes on $f^{\prime}\left( \eta \right)$ and thermal $\theta \left( \eta \right)$ profiles are shown in Fig. 3, against *m*, and the flow rate increases the temperature drop. A higher wedge angle causes this inclination boosts fluid velocity, which forces the boundary layer's thickness to drop and the temperature to elevate. The larger value of the Maxwell fluid parameter $\left( \lambda \right)$ causes the fluid motion to grow and declines the heat transfer, as publicized in Fig. 4. As $\lambda$ it climbs, the resistance forces between the fluid particles and the surface of the wedge fall, resulting in insufficient resistance to fluid motion which rises in the flow rate and declines the heat transfer rate.

Figure 5 depicts the behaviour of velocity profiles in reaction to deviations in Grashof $\left( {\beta_{1} } \right)$ and nonlinear Grashof numbers $\left( {\beta_{2} } \right).$ The correlation between buoyancy and frictional forces is referred to as the Grashof number; therefore, raising the values of $\beta_{1}$ and $\beta_{2}$ reducing the viscosity behaviour of nanoparticles, resulting in a slowdown in resistive forces to fluid motion. The fluid's velocity increases in this physical phenomenon, as demonstrated in Fig. 5. Figure 6 depicts how increasing levels of the porosity parameter (*Kp*) and the Darcy law parameter (*Fr*) slow down fluid velocity. When *Kp* and *Fr* climbed, the capacity of pore space enhanced, causing additional resistance to liquid motion and reducing the flow rate of the fluid. The velocity slip $\left( \gamma \right)$ and Biot number $\left( {{\text{Bi}}} \right)$ imprinted in Fig. 7 represent the fluid flow and temperature profiles. As demonstrated, the flow rate and thermal effects are enhanced by both velocity slip and Biot number. Velocity slip is expected to intensify fluid motion by inducing additional disruption. The reason for this is that as the velocity slip increases, the velocity of the fluid also increases. This increase in velocity results in greater applied forces, which push the expanding wedge and transfer energy to the liquid. But, it has been reported that hybrid nanofluids exhibit higher velocities than Maxwell fluids due to their improved thermophysical properties. So the ${\text{Bi}}$ is used to calculate heat transfer rate; we can conclude that ${\text{Bi}}$ has a direct relationship with thermal efficiency. Physically, elevating the values of the ${\text{Bi}}$ improves the thermal proficiency of the fluid, causing an upsurge in the heat transmission rate. The impact of the thermal parameter (*Nr*) and temperature ratio parameter $\left( {\theta_{r} } \right)$ on the thermal profile is demonstrated in Fig. 8.

An increase in $\theta_{r}$ and *Nr* results in an increase in the temperature field. Substantially, a larger $\theta_{r}$ shows a superior thermal transformation among the wedge wall and the neighboring atmosphere. The presence of a radiative component increases the mobility of small particles by inducing collisions between unrelated moving particles and converting frictional energy into thermal energy. As a result, a hybrid nanofluid exhibits a higher temperature than a conventional fluid in both scenarios. The thickness of the thermal boundary layer is due to the consequential temperature variation. The presence of a radiative component increases the mobility of small particles by inducing collisions between unrelated moving particles and converting frictional energy into thermal energy. As a result, a hybrid nanofluid exhibits a higher temperature than a conventional fluid in both scenarios.

The effect of the Eckert number (Ec) and the heat source/sink parameter (H) on the heat flux is highlighted in Fig. 9. It is acknowledged that as the *Ec* and *H* go up, the heat transfer boosts. Similarly, as equated to a Maxwell fluid, a tri-hybrid nanofluid has a faster heat transmission rate. The temperature profile is also improved due to dissipation effects in the energy equation. The main reason is that higher Ec values convert mechanical energy into thermal energy. When heat is produced, energy is transferred from the wedge to the working fluid. This increases the thermal field in the boundary layer region close to the wedge. However, as we move farther from the wedge, the temperature profile gradually reduces to zero.

In Fig. 10, we can see the impact of nanoparticle concentration on the flow and thermal fields of fluid, hybrid nanofluids, and ternary hybrid nanofluids. As the concentration of nanoparticles increases, the velocity of the fluid decreases while the thermal field improves. This occurs because the higher volume fraction of nanoparticles causes the momentum and thermal boundary layers to become denser, leading to greater resistance in the fluid. As a result, the velocity decreases. However, the presence of nanoparticles also increases the thermal conductivity of the fluid, resulting in higher fluid temperature. Furthermore, the ternary hybrid nanofluid exhibits the lowest velocity and the highest heat transfer rate compared to nanofluid and hybrid nanofluid.

### Fuzzy results and discussion

The determined fuzzy temperature $$\left( {\theta \left( {\eta ,\,\sigma } \right)} \right)$$ is visualized in Fig. 11 using volume fractions $$\phi_{1} ,\,\,\,\phi_{2} \,\,{\text{and}}\,\,\phi_{3}$$ as the TFN [0, 5, 10%]. Four sub-plots for triangular M.F.s demonstrate the $$\theta \left( {\eta ,\,\sigma } \right)$$ varying values of $$\eta = 1, \, 2, \, 3, \, 4.$$ The M.F. of the $$\theta \left( {\eta ,\,\sigma } \right)$$ for $$\sigma {\text{ - cut}}$$ is on the vertical axis, while the $$\theta \left( {\eta ,\,\sigma } \right)$$ for different values of $$\eta$$ is on the horizontal axis. The values calculated for TFN $$\theta \left( {\eta ,\,\sigma } \right)$$ are not symmetrical in a triangle shape, but the fuzzy volume fraction is symmetrical and also a part of TFN. These differences may be due to the nonlinearity of the governing FDE. Furthermore, hybrid nanofluids were discovered to have a larger range than nanofluids, which results in the TFN being unable to accurately determine the hybrid nanofluid. In Fig. 11, nanofluids, $${\text{Al}}_{{2}} {\text{O}}_{{3}} {\text{/EO,}}$$$${\text{Cu/EO,}}$$$${\text{TiO}}_{{2}} {\text{/EO,}}$$ hybrid nanofluids $$\left( {{\text{Al}}_{{2}} {\text{O}}_{{3}} + {\text{Cu/EO}}} \right)$$ and ternary hybrid nanofluids $$\left( {{\text{Al}}_{{2}} {\text{O}}_{{3}} + {\text{Cu}} + {\text{TiO}}_{{2}} {\text{/EO}}} \right)$$ are compared using M.F. for different values of $$\eta .$$ There are four scenarios considered in these figures. The first scenario $$\phi_{1}$$ shows as TFN and $$\phi_{2} = 0$$ epitomized by blue lines. The second scenario $$\phi_{2}$$ shows as TFN and $$\phi_{1} = 0$$ epitomized by red lines. The third scenario shows the hybrid nanofluid with both $$\phi_{1}$$ and $$\phi_{2}$$ non-zero, epitomized by black lines. Ternary hybrid nanofluids $$\phi_{1} ,\,\,\,\phi_{2} \,\,{\text{and}}\,\,\phi_{3}$$ are non-zero, which represents red lines. The hybrid nanofluid performs better than both nanofluids because the temperature variance is larger in the hybrid nanofluid. In a ternary hybrid nanofluid, the thermal conductivities of $${\text{Al}}_{{2}} {\text{O}}_{{3}}$$, $${\text{Cu}}$$ and $${\text{TiO}}_{{2}}$$ are combined to achieve greater heat transfer physically. As the temperature of the fluid rises, so does its thermal conductivity. It's worth noting that the nanoparticles and temperature play a role in increasing thermal conductivity in nanofluids. The increase in temperature caused by the materials' intrinsic thermal conductivity phenomenon improves thermal conductivity. The thermal conductivity of most nanomaterials increases as the temperature rises, transferring more energy between the particles in the standard-base fluid. The temperature of the fluid rises, causing molecular motions to increase, which improves energy transmission.

The results for hybrid nanofluids $\left( {{\text{Al}}_{{2}} {\text{O}}_{{3}} + {\text{Cu/EO}}} \right)$ and ternary nanofluids $\left( {{\text{Al}}_{{2}} {\text{O}}_{{3}} + {\text{Cu}} + {\text{TiO}}_{{2}} {\text{/EO}}} \right)$ are achieved. Table 4 displays the coefficient of skin friction and temperature fluctuations. Table 4 shows that skin friction is elevated via Mh*, m, Nr, Ec, *$\beta_{1}$ and $\beta_{2}$ while the decline via *Kp* and *Fr*. Table 4 demonstrates the numerical results for the surface heat transfer rate. The increase in Mh, m, $\beta_{1}$ and $\beta_{2}$ the surface heat transfer rate for hybrid nanofluid and ternary nanofluid is prominently amplified, and the opposite results were found for rising *Kp, Fr, Nr,* and Ec. Observations suggest that the wall heat transfer rate is higher in a ternary nanofluid than in a hybrid nanofluid.

**Table 4:** Numerical results of ternary nanofluid and hybrid nanofluid for $Cf_{x}$ and ${\text{Nu}}_{x} .$

Mh	*kp*	*Fr*	*m*	*Nr*	Ec	$\beta_{1}$	$\beta_{2}$	Ternary hybrid nanofluid		Hybrid nanofluid	
								$Cf_{x}$	$Nu_{x}$	$Cf_{x}$	${\text{Nu}}_{x}$
0.1								1.4813617	0.7238273	1.3354321	0.6629934
0.2								1.5800616	0.7480380	1.4318217	0.6881096
0.3								1.6772433	0.7722202	1.5266534	0.7131605
	0							1.9953789	0.8649554	1.8175022	0.8021721
	0.1							1.8673769	0.8208693	1.7119769	0.7635208
	0.2							1.8028922	0.7975733	1.6149688	0.7292884
		0						1.9408465	0.8482189	1.8505440	0.8196600
		0.1						1.8673769	0.8208693	1.7763973	0.7891355
		0.2						1.8028922	0.7975733	1.7119769	0.7635208
			0.1					1.6062909	0.7355098	1.5388373	0.7022927
			0.2					1.7195612	0.7717105	1.6383504	0.7378857
			0.3					1.8028922	0.7975733	1.7119769	0.7635208
				0.1				1.7889124	0.8871146	1.6945150	0.7967321
				0.3				1.7962520	0.8378307	1.7045150	0.8067321
				0.5				1.8028922	0.7975733	1.7119769	0.7635208
					0			1.8192235	0.6746530	1.7297125	0.6355862
					0.1			1.8277141	0.6130720	1.7389590	0.5713825
					0.2			1.8364230	0.5513847	1.7484617	0.5069854
						0		1.7497042	0.7888631	1.6543778	0.7530693
						0.1		1.7766224	0.7932643	1.6835818	0.7583571
						0.2		1.8028922	0.7975733	1.7119769	0.7635208
							0.1	1.7972346	0.7968564	1.7039876	0.7623880
							0.2	1.7986522	0.7970360	1.7055912	0.7626153
							0.3	1.8000677	0.7972153	1.7071920	0.7628422

## Conclusions

A comprehensive analysis of 2D Darcy's Forchheimer Maxwell fuzzy ternary hybrid nanofluid flow towards a vertical Riga wedge with nonlinear mixed convection and thermal radiation is inspected. The following are some intriguing findings that were drawn from the current work:

- The ternary hybrid nanofluid shows better heat transfer than conventional Maxwell fluid.
- Darcy-Forchheimer and porous media decline the flow due to nanomaterials being slower.
- The heat transfer has amplified when the Eckert number, nonlinear thermal radiation, and heat source improve.
- Nonlinear convection and nanoparticle volume fractions reduced the fluid velocity, whereas the opposite behavior is observed for Mh*, *$\gamma$ and* m*.
- The skin friction upsurges with M*h, m, *$\beta_{1}$ and $\beta_{2}$ but the rising values of *Kp* and *Fr* cause to diminish the skin friction.
- The nanoparticle's volume fraction is said to be the triangular fuzzy number. According to the fuzzy analysis, ternary hybrid nanofluid shows maximum heat transfer compared to the hybrid nanofluid and nanofluid.
- With developing values of Mh, *m*, $\beta_{1}$ and $\beta_{2}$, progressive heat transfer rate, it is inversely proportional to *Kp* and *Fr*.

## Data Availability

The datasets generated and/or analysed during the current study are available in this manuscript.

## List of symbols

$v$ and $u$: Velocity components (m/s)
*T*: Fluid temperature (K)
$$T_{f}$$: Surface temperature (K)
$$T_{\infty }$$: Ambient temperature (K)
$$\psi$$: Wedge angle
$$\theta_{r}$$: Temperature difference
$$s_{1} ,$$$$s_{2}$$ and $$s_{3}$$: Solid nanoparticles of $${\text{Al}}_{{2}} {\text{O}}_{{3}}$$, Cu and $${\text{TiO}}_{2}$$
*g*: Gravity $$\left( {{\text{m}}/{\text{s}}^{2} } \right)$$
$H$: Heat source parameter
$$\Pr$$: Prandtl number
$$Nr$$: Thermal radiation
$\phi_{1}$, $\phi_{2}$ and $\phi_{3}$: The volume percentages of ${\text{Al}}_{{2}} {\text{O}}_{{3}}$, Cu and ${\text{TiO}}_{2}$ nanomaterials
$Cf_{x}$: Local skin friction
$m$: Hartree pressure gradient
$v_{f}$: Kinematic viscosity of fluid $\left( {{\text{m}}^{2} /{\text{s}}} \right)$
$J_{0}$: Current density of electrodes
$$kp$$: Porosity parameter
$\mu_{trhnf}$: Dynamic viscosity of trihybrid nanofluid $({\text{kgm}}^{2} /{\text{s}})$
$\left( {\rho C} \right)p_{trhnf}$: Heat capacity of trihybrid nanofluid
$k_{trhnf}$: Thermal conductivity of trihybrid nanofluid
$\rho_{trhnf}$: Density of trihybrid nanofluid $\left( {{\text{Kg}}/{\text{m}}^{3} } \right)$
$$\eta$$: Similarity variable
$$\omega$$: Stream function
$u_{sv}$: Free stream velocity (m/s)
$a_{h}$: Non-dimensional parameter
$$\gamma$$: Slip parameter
$${\text{Bi}}$$: Biot number
$\lambda$: Maxwell fluid parameter
${\text{Mh}}$: Modified Hartmann number
${\text{Nu}}_{x}$: Local Nusselt number
$$\theta \left( \eta \right)$$: Dimensionless temperature
$$f^{\prime}\left( \eta \right)$$: Dimensionless velocity
$\beta_{1}$ and $\beta_{2}$: Grashof and nonlinear Grashof numbers
$Fr$: Darcy law parameter
